# Four Curious Cases of Closed-Incision Negative Pressure Therapy (ciNPT)

**DOI:** 10.7759/cureus.8193

**Published:** 2020-05-18

**Authors:** Sarah Huan, Luke Tay

**Affiliations:** 1 Hand Surgery, Singapore General Hospital, Bukit Merah, SGP; 2 Vascular Surgery, Singapore General Hospital, Bukit Merah, SGP

**Keywords:** closed incision negative pressure therapy, post-operative management, postoperative wounds, wound healing, surgical wound complications, vascular surgery, lower limb wounds

## Abstract

Post-operative wound complications after infrainguinal vascular surgery can lead to a significant decrease in a patient’s quality of life. The main complications are surgical site infection, wound dehiscence, seroma, hematoma, delayed healing and/or poor scarring. Closed-incision negative pressure therapy (ciNPT) in particular has recently been suggested as a method of prophylaxis against these surgical site complications when applied over a closed wound. In our unit, it has generally been used for long groin and abdominal surgical wounds.

We describe here four relatively unusual cases of our experience using ciNPT. In our series, ciNPT has been used to good effect on small eccentrically shaped wounds and non-resolving groin hematomas. We have also used it in tandem with intra-cavity Vacuum Assisted Closure (VAC) (KCI, Acelity, San Antonio, TX) dressings for the management of partially closed wounds to reduce risk of infection and dehiscence. Additionally, it has shown good analgesic effects in our patient post-operatively.

Hence, we suggest that the paradigm of ciNPT can be expanded to include these circumstances to improve wound healing and decrease risk of post-operative complications. Further research into the overall clinical benefits and cost-effectiveness will also be helpful.

## Introduction

The incidence of postoperative wound complications after infra-inguinal vascular surgery has been reported to be as high as 44% [1]. These can result in a significant decrease in the patient’s quality of life. Diabetes mellitus, smoking, obesity, and end-stage renal failure have been reported to increase the risk of developing complications in peripheral vascular surgery [2-4]. Negative pressure therapy, which is usually used in open wounds to stimulate granulation and reduce edema, has been recently suggested as a method of prophylaxis against surgical site complications when applied over a closed surgical wound [5].

The concept of closed-incision negative pressure therapy (ciNPT) was first realized commercially in 2010 with the launch of the Prevena^TM^ Incision Management System (Acelity, San Antonio, US). This was followed quickly by the launch of the Pico^TM^ system (Smith & Nephew, St. Petersburg, PL) in 2011. Initial adoption was slow owing to skepticism regarding the utility of applying negative pressure to closed wounds, coupled with the upfront cost of the dressings. More recently, increasing evidence has emerged supporting reductions in wound complications in the breast, orthopedic, obstetrics, and vascular surgical procedures [6-9].

Our unit is a vascular surgical department in a tertiary hospital in Singapore which performs limb revascularization and wound care. We have been using ciNPT for the last three years in patients with long groin and abdominal wounds from femoral endarterectomies and bypass operations.

Of late, we have been applying it in more unusual circumstances. We describe herein our experiences with four cases of ciNPT which we consider fairly novel, in the hopes that we may help to expand the paradigm for ciNPT further.

## Case presentation

Case 1

A 60-year-old male with a forty pack-year smoking history and a past medical history of type 2 diabetes mellitus complicated by nephropathy, retinopathy, and previous cerebrovascular accident with good functional recovery was admitted with an acutely swollen, painful right big toe with an associated ulcer and cellulitis extending into the forefoot.

**Figure 1:**
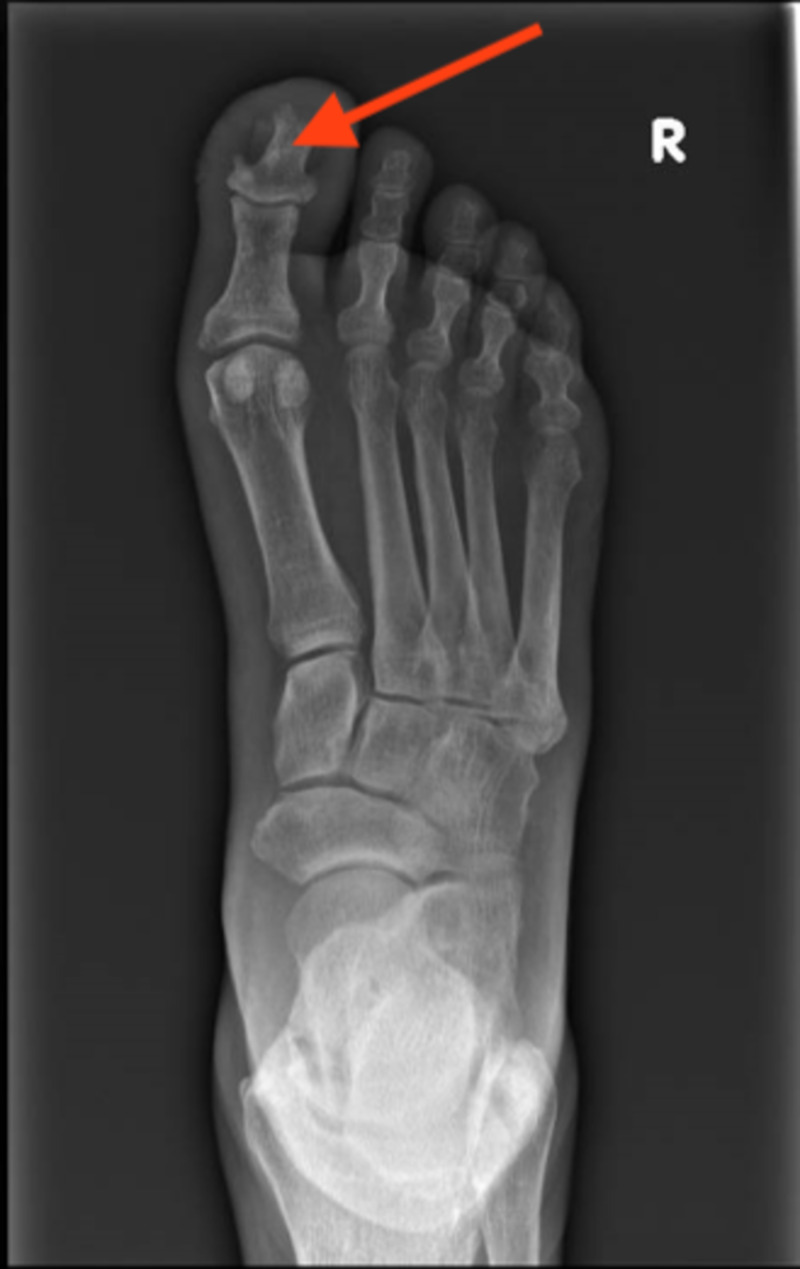
X-ray showing erosion of distal phalanx of the right big toe

On examination, there was erythema and warmth over the right foot dorsum and lower shin, with a deep 5 mm diameter ulcer on the plantar surface of the right big toe with serous discharge. The right lower shin was shiny with darkening of skin and xerosis was noted. Distal pulses were palpable. An X-ray of the foot showed osteomyelitic erosion of the distal phalanx of the big toe with surrounding soft tissue swelling (Figure 1).

A duplex arterial scan showed multiple stenoses of the superficial femoral artery (SFA), popliteal, posterior tibial artery (PTA) and peroneal arteries and long segment occlusion of the anterior tibial artery for which he underwent right lower limb angioplasty to the SFA, PTA and peroneal arteries with terminalization of the right big toe.

**Figure 2:**
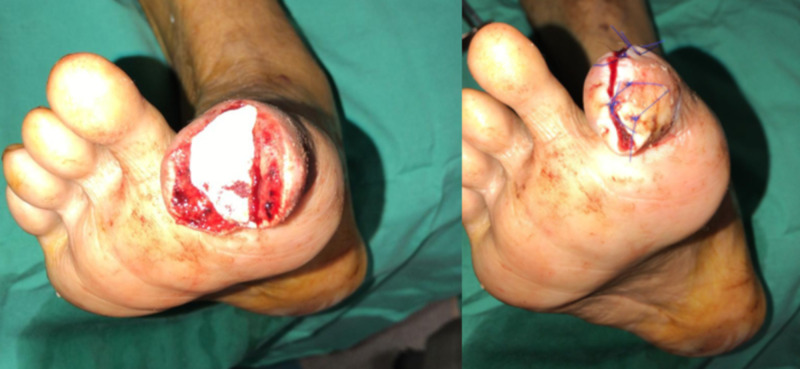
Excision of the distal phalanx and closure over Promogran Prisma dressing Left – wound before closure. Right – wound after closure.

Angioplasty to the SFA, popliteal, PTA, and peroneal arteries was performed with concurrent excision of the terminal phalanx and primary closure of the skin envelope over a Promogran^TM^ (Acelity and KCI Headquarters, San Antonio, TX) and application of ciNPT using the Prevena^TM^ Customizable^TM^ (Acelity, San Antonio, US) system (Figure 2).

**Figure 3:**
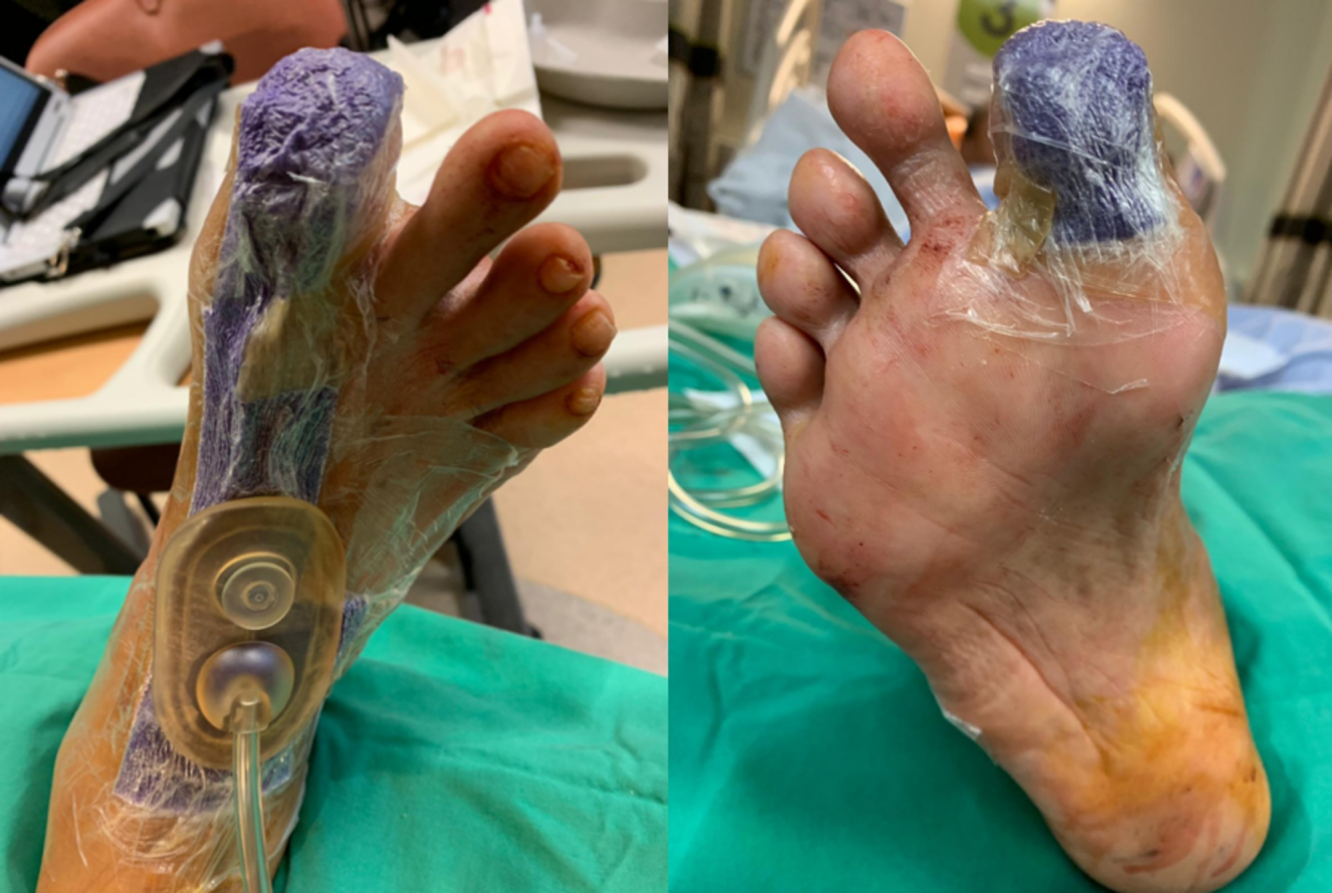
Technique of applying Prevena to big toe wound closure Left – dorsal view. Right – plantar view.

Application was extremely challenging as the terminalized digit presented a small and eccentrically contoured surface to which the dressing would have to seal. The wound was first cleaned and draped, its length measured and the Prevena Customizable dressing cut to size accordingly. Excess dressing length was put away to use for bridging of the TRAC pad (KCI, San Antonio, TX) onto the dorsum of the foot. The hydrocolloid strips were applied to both ends of the incision and along its length and the drape was cut into small thin strips. The cut customizable dressing was then applied to the incision, starting from the volar aspect around to the dorsal aspect of the toe and then onto the dorsum of the foot, being careful not to dislodge the previously placed hydrocolloid strips. The dressing was secured with the pre-cut drapes, starting from distal to proximal and over the dorsum of the foot, making sure it stuck down properly onto the hydrocolloid stripes (especially in the web space). A hole was cut in the drape over the dorsal foot and the Prevena TRAC pad applied. The dressing was then connected to the adaptor and then the ActiVAC (KCI Technology, San Antonio, TX) machine. The final product can be seen in Figure 3.

**Figure 4:**
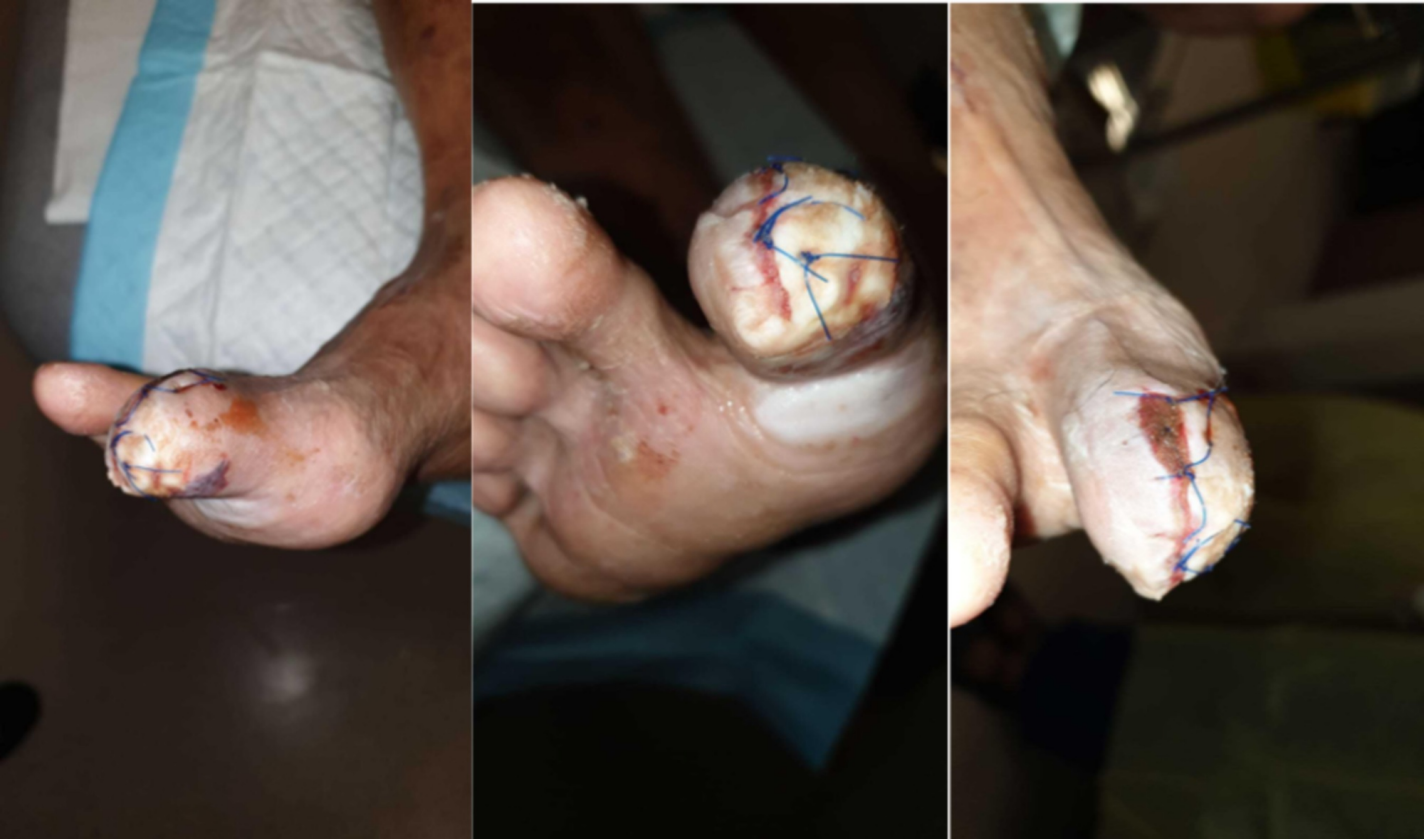
At one week, the wound remained clean and viable; left, middle and right panels show different views of the wound

He was able to ambulate the following day and was discharged. The Prevena dressing was removed after one week. The wound was clean and uninfected, with some minimal maceration of the skin. The wound was dressed with daily povidone iodine gauze (Figure 4).

**Figure 5:**
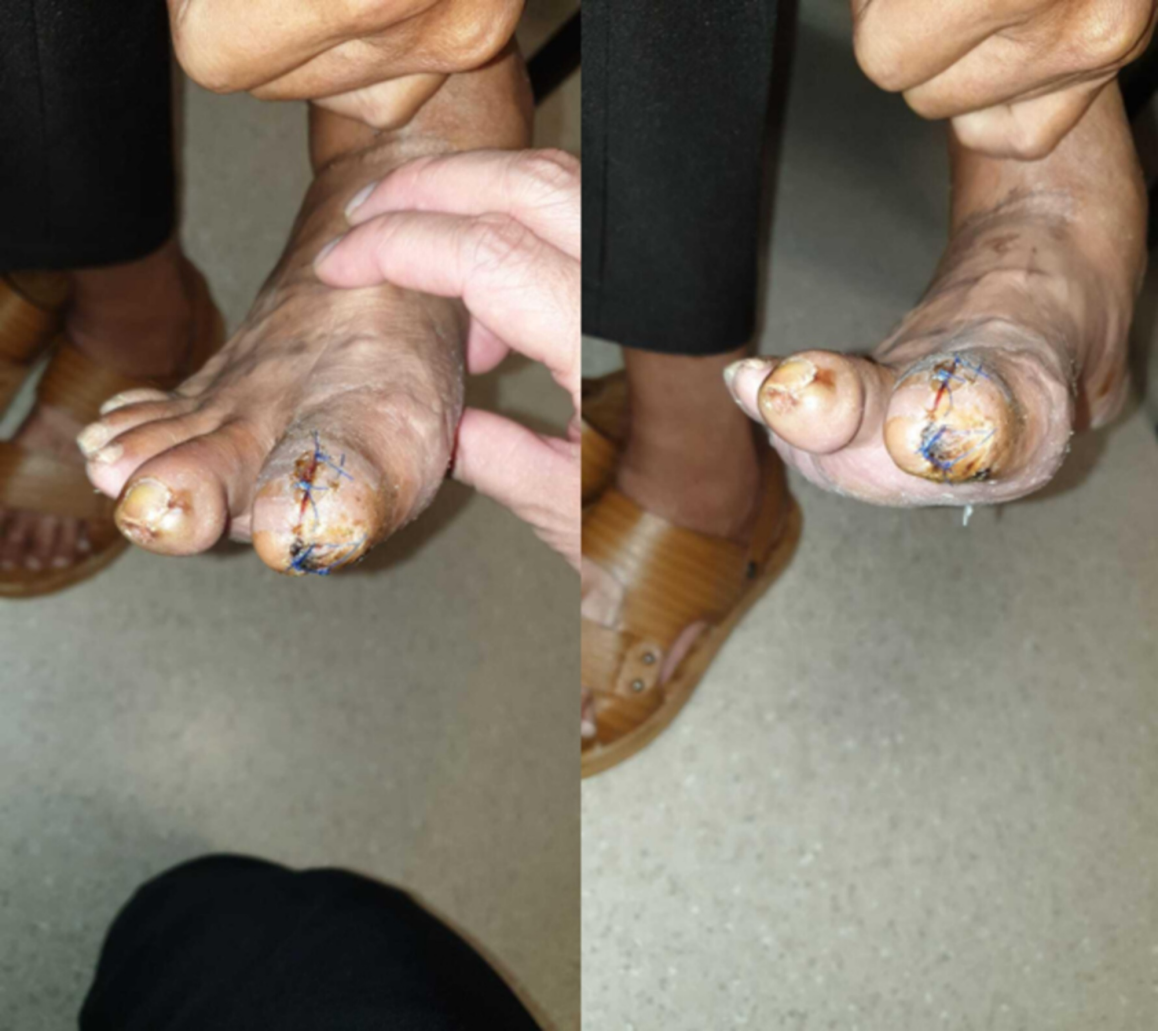
At two weeks, the wound was healing well; left and right show different views of the same wound

A week later, the skin condition had improved and the wound was healing well (Figure 5).

**Figure 6:**
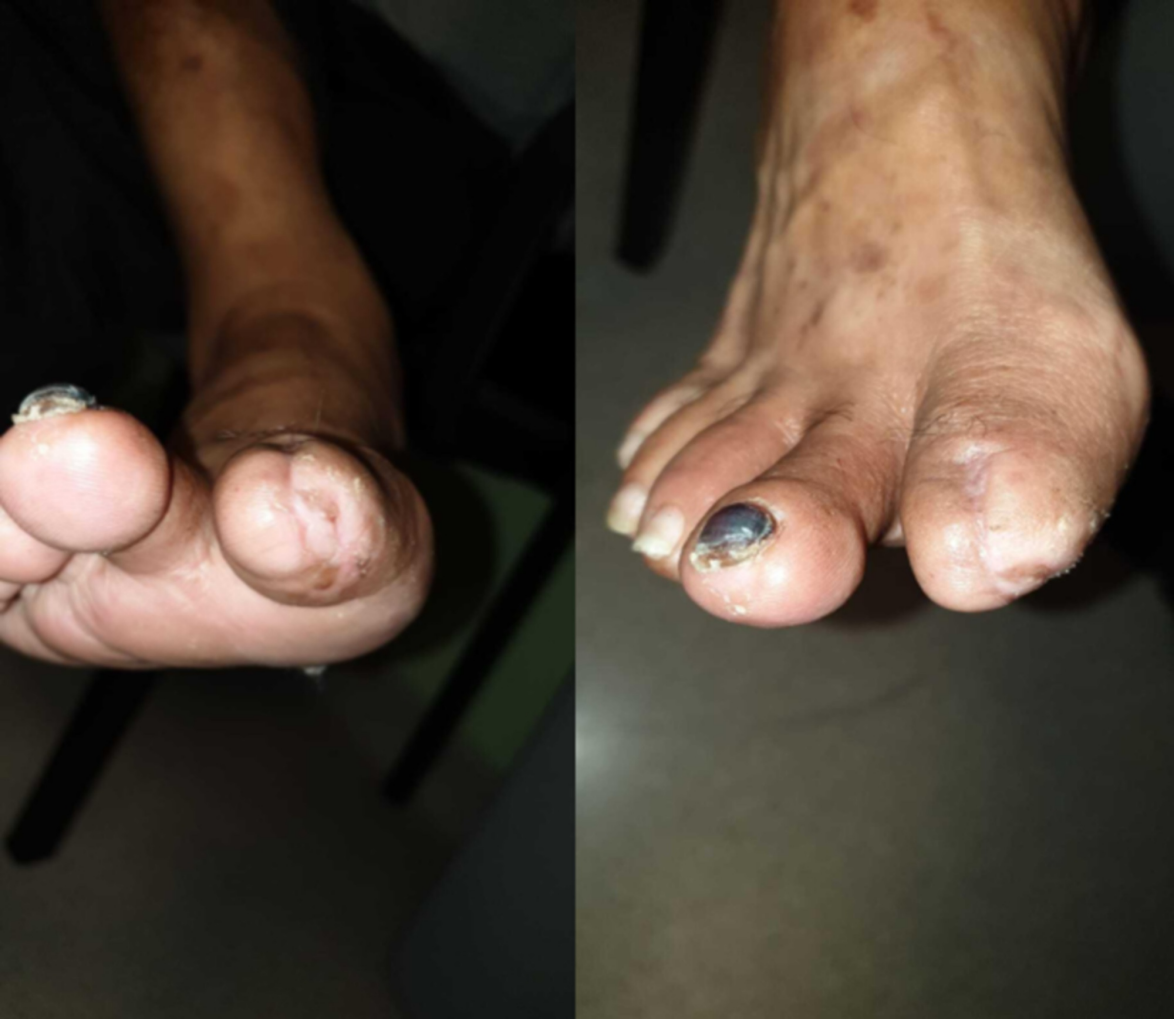
Complete healing at two months; left and right show different views of the same wound

On follow-up two months later, the wound had healed completely (Figure 6). In comparison to lower limb wounds, in other vascular patients in our institution, this was a much shorter time interval.

Case 2

**Figure 7:**
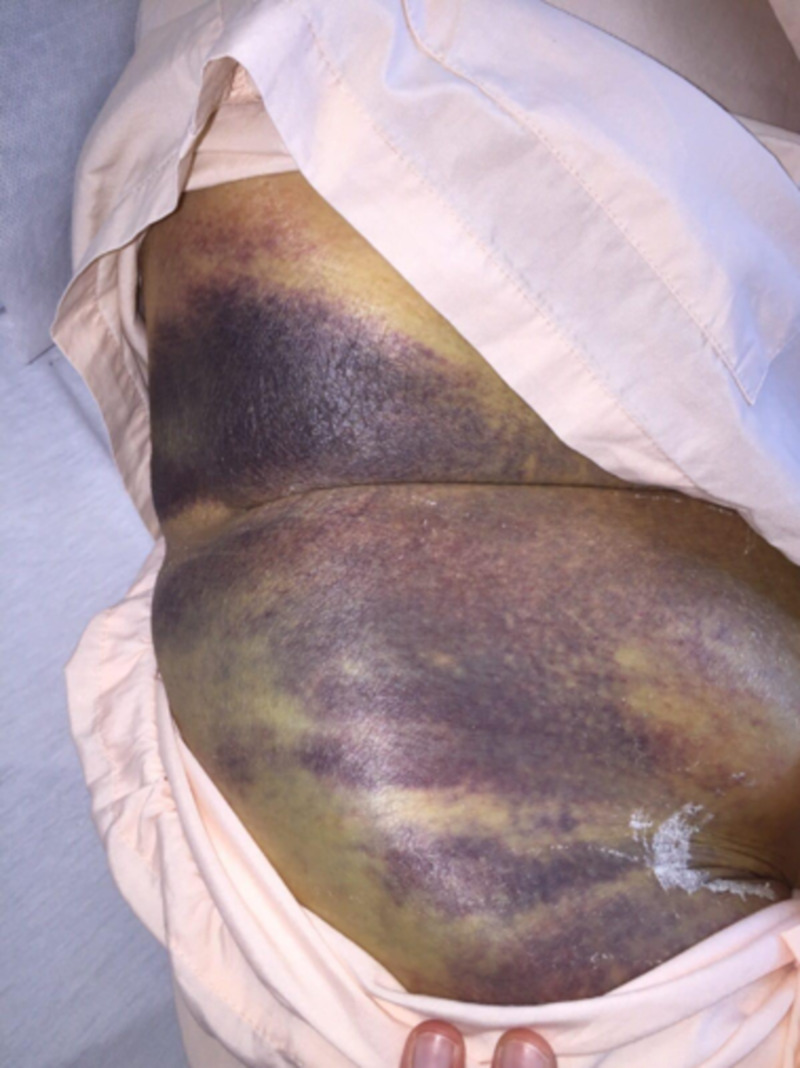
Extensive right groin ecchymosis with hematoma

A 69-year-old female with a history of type 2 diabetes mellitus, atrial fibrillation on apixaban, and chronic kidney disease was referred to our department for a large right groin hematoma with extensive ecchymosis complicated by hypotension after a percutaneous coronary intervention with drug-eluting stent (DES) (Figure 7). An urgent angiogram showed active bleeding from a needle injury to a branch of the SFA which was successfully treated with a 7 x 22 mm atrium balloon-expandable (BE) covered stent to the SFA, after attempts to cannulate the branch failed. She was transferred to the Coronary Care Unit thereafter.

In light of the DES, she was started on dual antiplatelet therapy and a one-week course of co-amoxiclav, with daily iodine-soaked gauze dressing to the groin puncture site. Her recovery was otherwise uneventful during this admission and she was transferred to a community hospital for rehabilitation thereafter.

She was readmitted to us five weeks later after the previous puncture wound started to ooze blood. A focused duplex ultrasound scan of the femoral artery excluded a recurrent pseudoaneurysm but sized the hematoma at 15.21 x 8.37 x 4.83 cm.

She was started on antibiotics and assessed by plastic surgery who felt that the overlying skin was non-viable requiring surgical debridement and evacuation of the hematoma. However, she adamantly refused surgery, so we attempted to manage the fluid discharge with a stoma bag over the sinus. The drainage was constant and fairly voluminous (200 ml/day) and the position of the sinus close to the groin crease was suboptimal for sealing, both factors in combination causing the stoma adhesive to be ineffective with persistent leakage around the stoma, which in turn caused her great distress. We then placed a 13 cm Prevena^TM^ Peel & Place^TM^ system over the sinus which managed to seal the leakage effectively and drained the fluid from the Prevena dressing in a closed fashion into an InfoVACTM machine.

**Figure 8:**
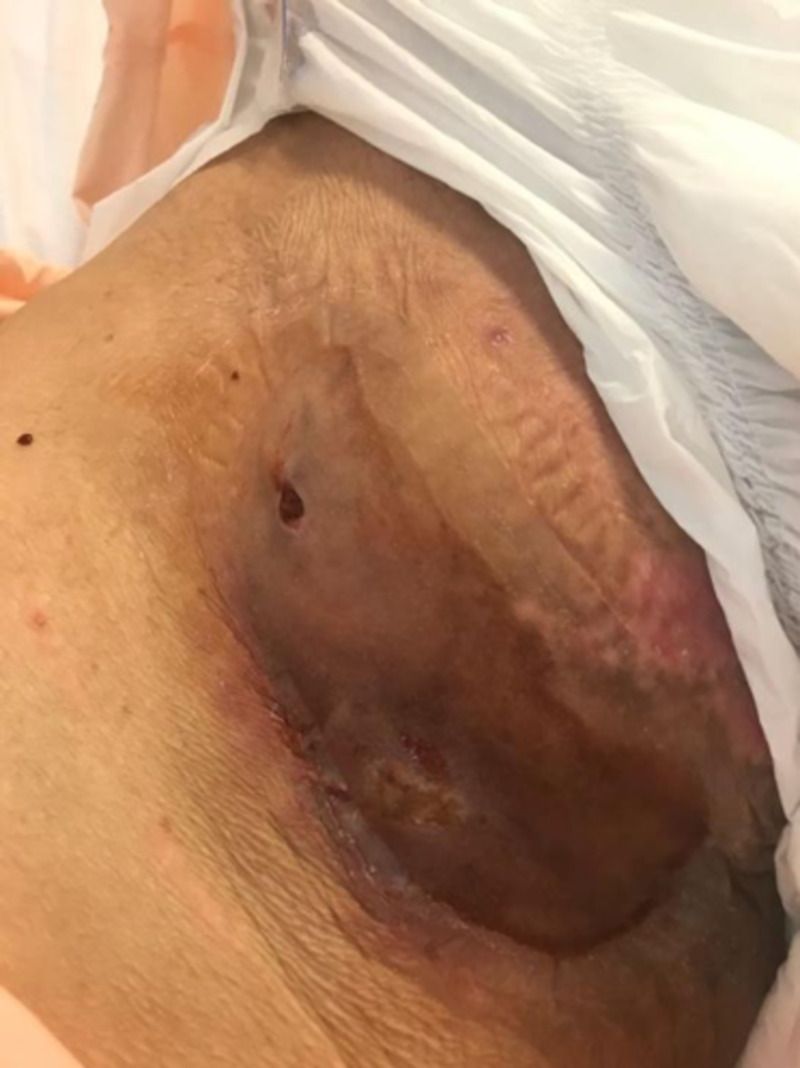
One week after placement of Prevena as fluid management system

At one week, the skin remained unhealthy-looking but the rate of drainage had decreased to about 50 ml/day (Figure 8). Prevena was reapplied for fluid management and attached to an ActiVAC machine and the patient was discharged.

**Figure 9:**
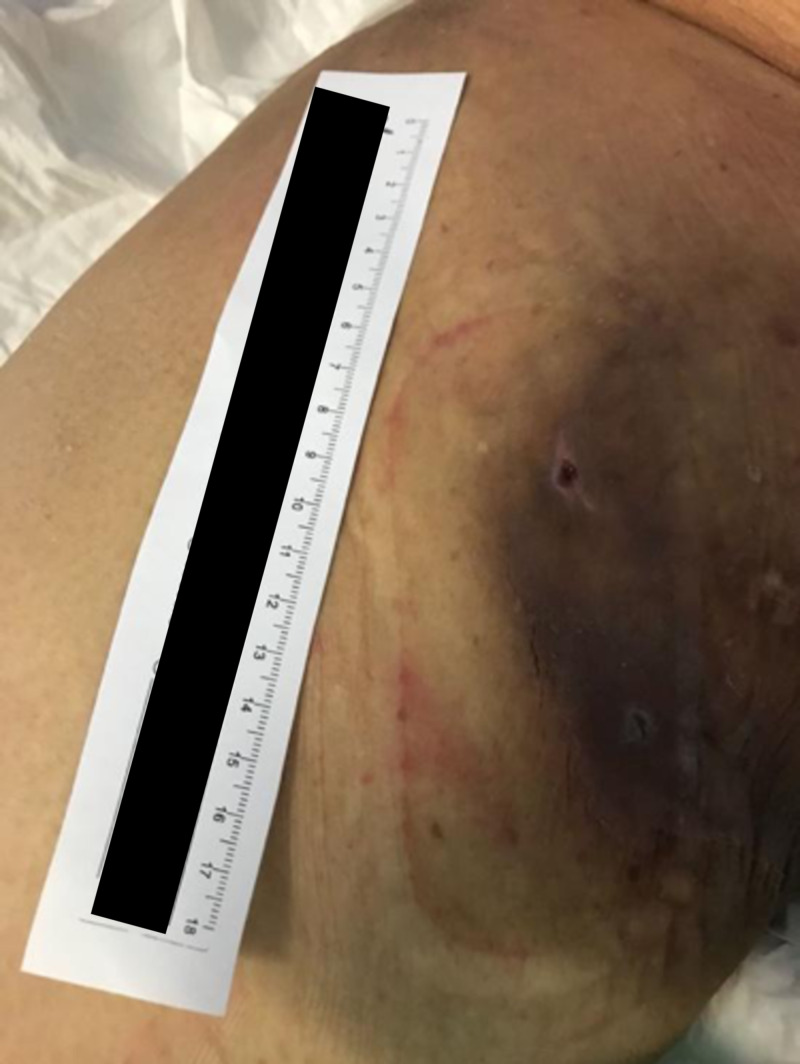
Two weeks after placement of Prevena demonstrating reduction in size of hematoma and improvement in overlying skin

On inspection a week later, the hematoma was smaller at 9 x 4 cm, and no longer fluctuant (Figure 9). As fluid drainage had ceased, the Prevena was discontinued.

**Figure 10:**
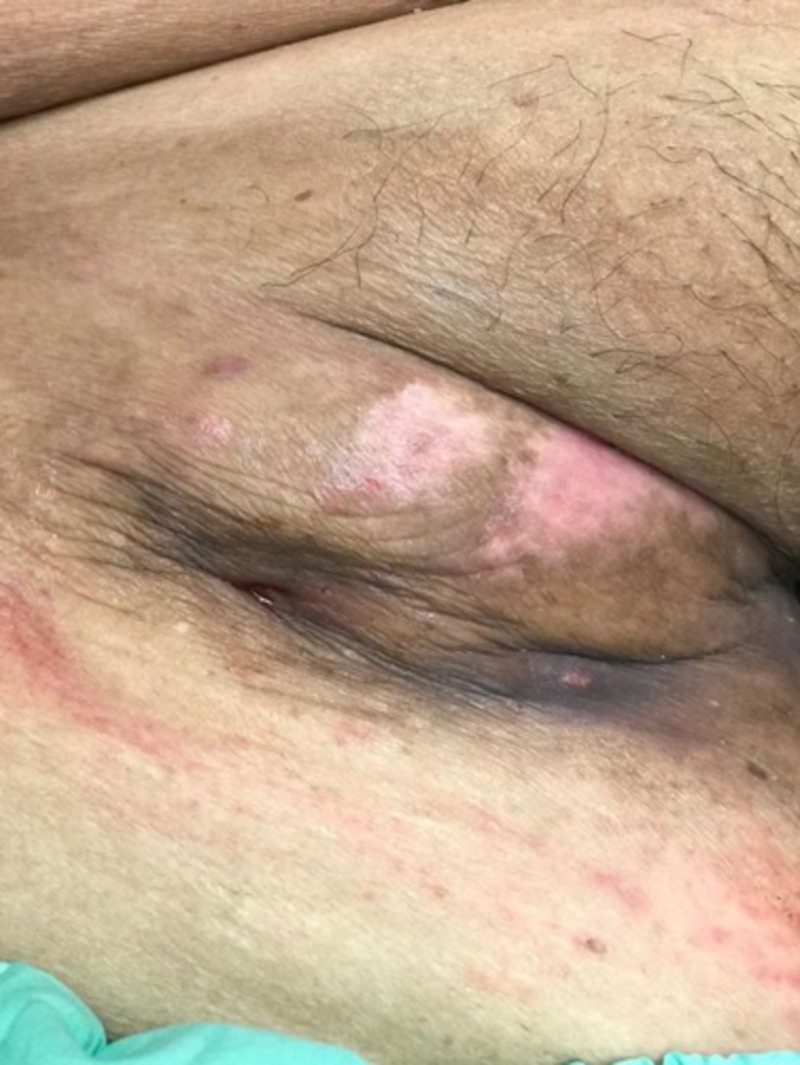
Four weeks from first application, with recovery of overlying skin and resolution of fluid drainage

Two weeks later, which was four weeks from the time of first application, the previously-deemed nonviable skin now appeared healthy although somewhat scarred (Figure 10). She was given an open appointment.

Case 3

A 62-year-old female with a past medical history of type 2 diabetes mellitus complicated by peripheral vascular disease, end-stage renal failure (on hemodialysis), and ischemic heart disease was originally admitted to our service for left foot wet gangrene. The gangrene was extensive and her foot could not be salvaged so she underwent an above knee amputation (AKA). She was discharged to a community hospital for rehabilitation.

Four months postoperatively she was readmitted for infection and breakdown of the AKA wound with pain, bleeding, and a foul-smelling serosanguinous discharge.

**Figure 11:**
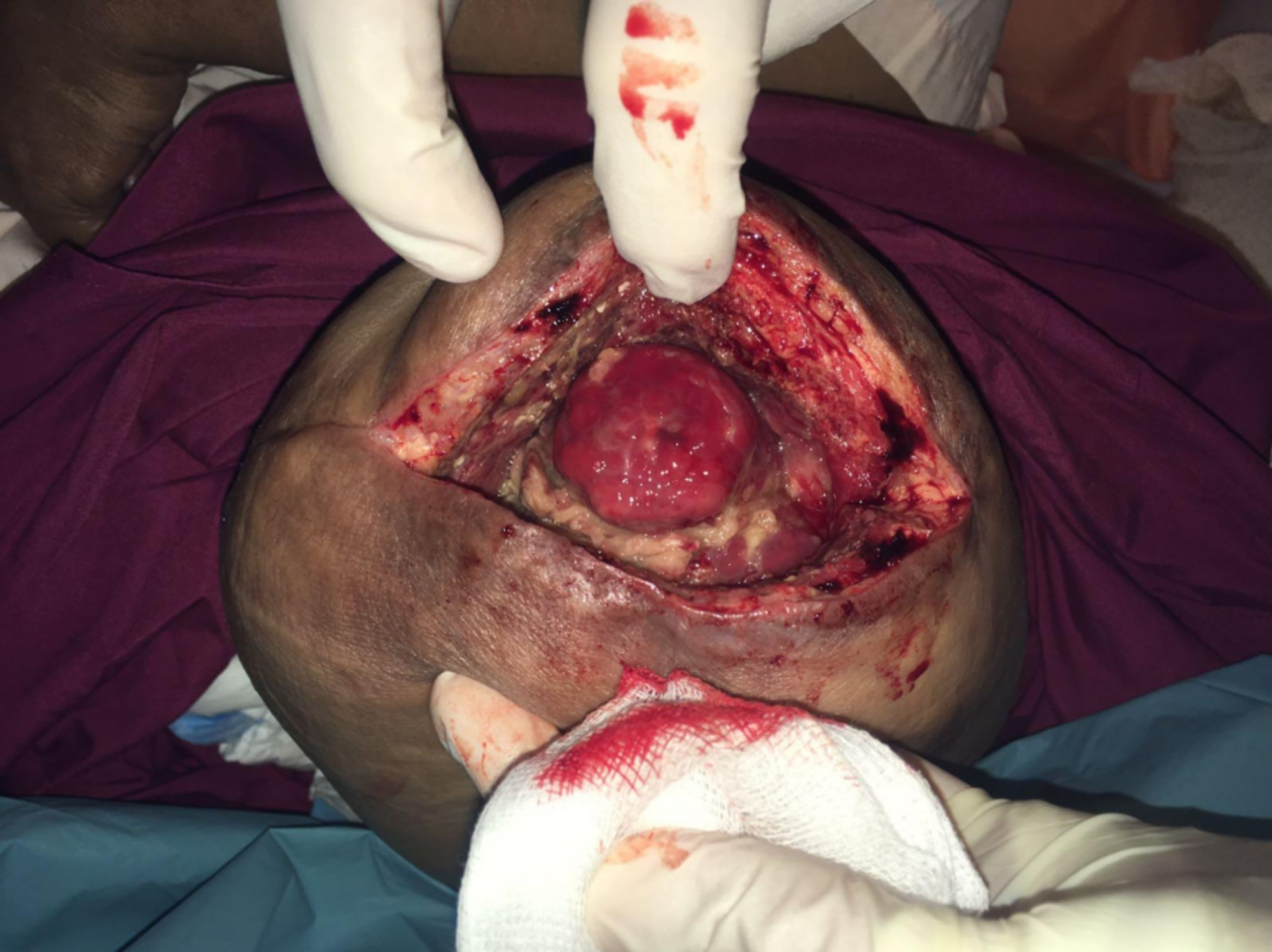
Infected above knee amputation wound after exploration and debridement

She was started on intravenous co-amoxiclav and underwent exploration and debridement of the AKA stump the following day. The wound cavity measured 15 x 20 cm with copious infected old hematoma, dirty necrotic fat and muscle, and exposed bone with overlying unhealthy granulation (Figure 11). The wound was opened and curetted, and washout with Pulsavac® (Zimmer Inc, Warsaw, IN) lavage was performed. The wound was dressed with a Vacuum-Assisted Closure (VAC) (KCI, Acelity, San Antonio, Texas) dressing. Wound cultures grew methicillin-resistant Staphylococcus aureus.

**Figure 12:**
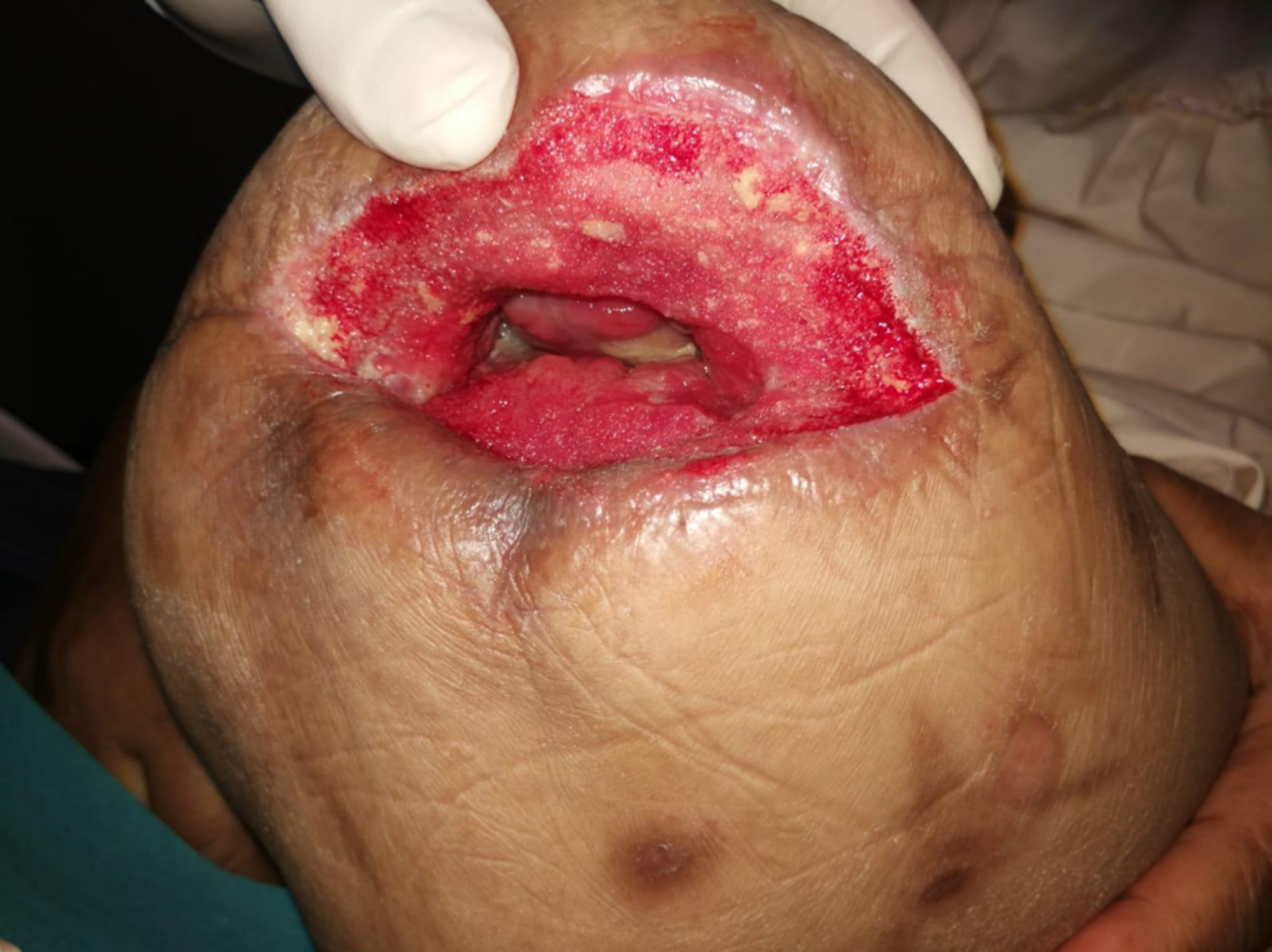
Two weeks of VAC therapy after index operation, wound clean and granulating VAC: Vacuum Assisted Closure.

Her wound gradually improved over the course of the next two weeks with regular VAC dressing changes and bedside debridements, with an increase in granulation and reduction in slough (Figure 12).

**Figure 13:**
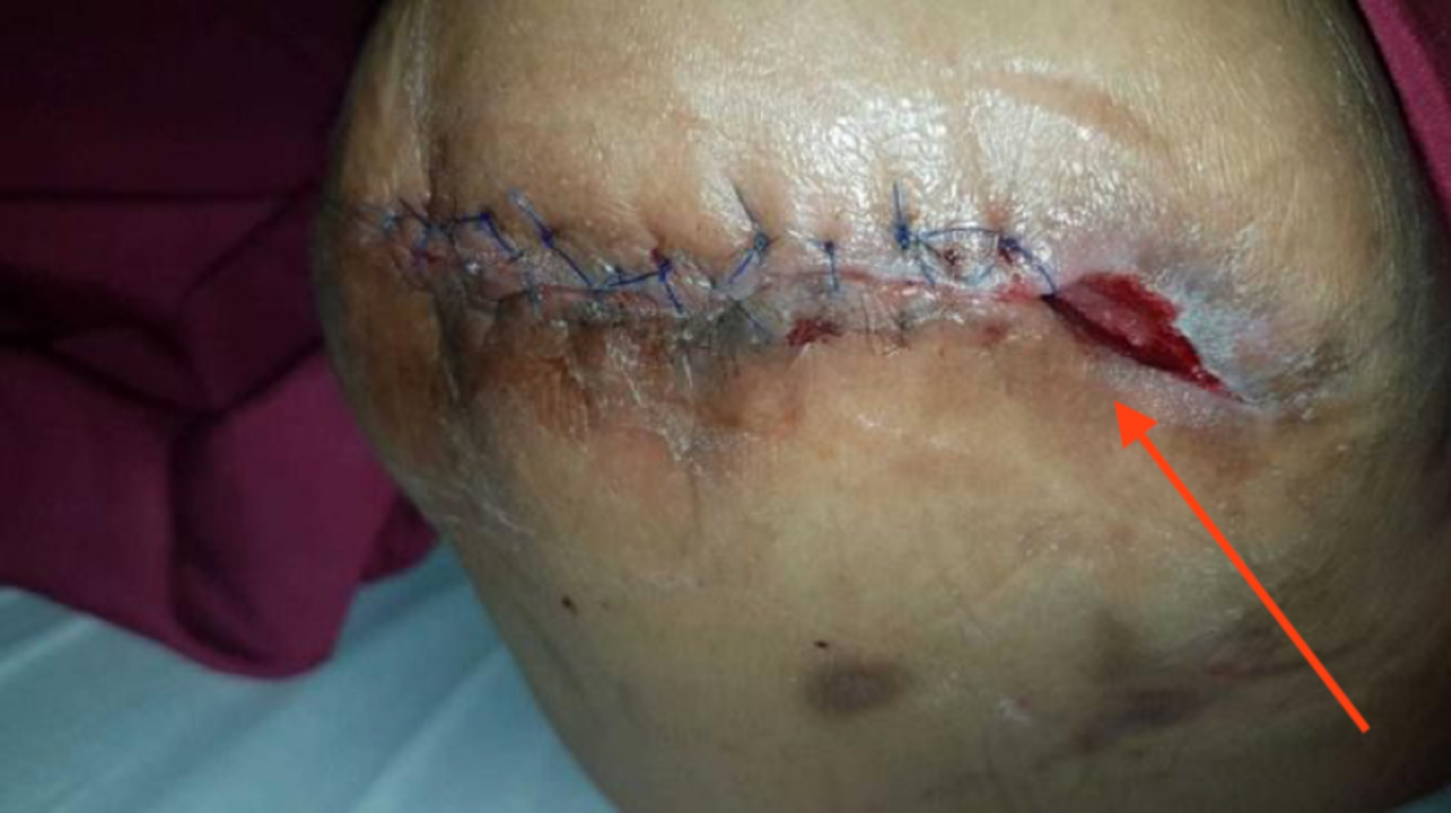
Demonstrating the wound appearance after secondary suture, with an exudative lateral cavity which could not be drawn together at this point

She subsequently underwent secondary suture to the AKA stump, however the lateral 1 inch of the stump was too retracted to close and remained slightly sloughy with persistent exudate, so it was left open. There was a long medial tract beneath the length of the sutured skin (Figure 13).

As her wound was complicated, we attempted to create a “hybrid” negative pressure dressing aimed at conferring the benefits of both intra-cavity traditional negative pressure wound therapy (tNPWT) as well as ciNPT as follows.

**Figure 14:**
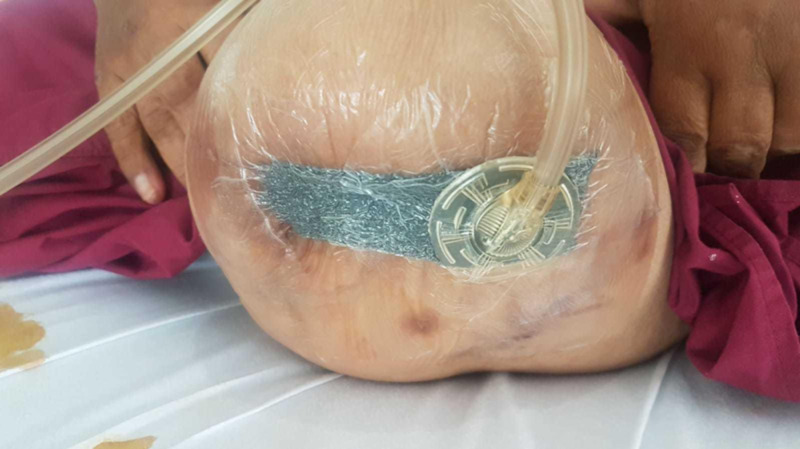
The external appearance after application of combination home-made Prevena plus intra-cavity VAC dressing VAC: Vacuum Assisted Closure

The lateral cavity and tract under the closed suture-line was packed with a long wedge of VAC foam, and a “home-made” Prevena created by lining both edges of the sutured portion of the wound with the VAC drape, leaving a small gap along the length of the suture line. The conventional VAC foam was cut into a strip and laid over the suture-line and the foam-packed cavity, the wound sealed with overlying VAC drape and the suction head applied, resulting in an “all-in-one” combination of intra-cavity VAC to promote granulation and reduce exudate, and superficial negative pressure to the suture-line to recreate the benefits of ciNPT (Figure 14).

**Figure 15:**
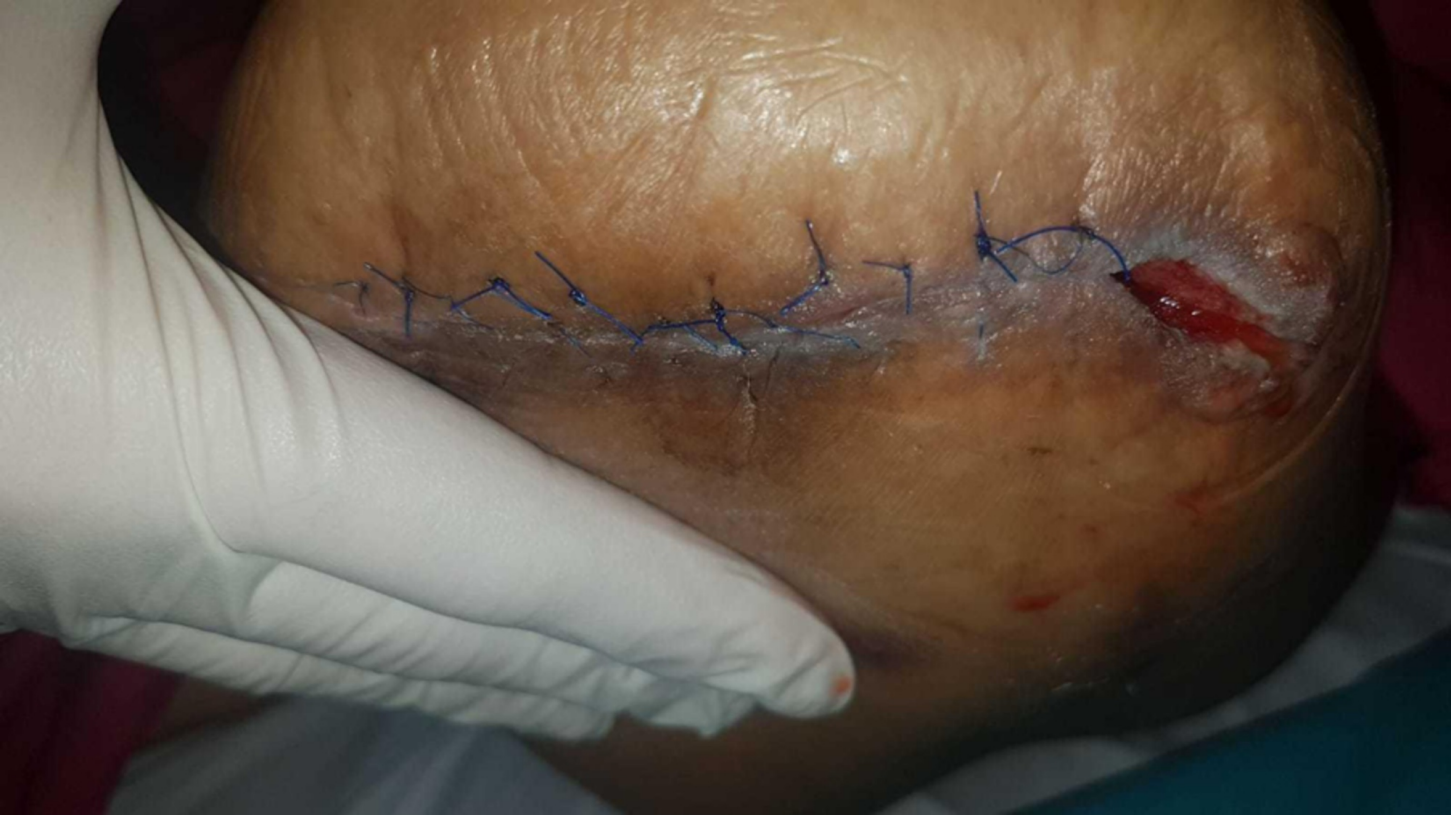
Demonstrating skin union of the sutured portion at one week, with contraction of the lateral wound

This dressing was changed every three to four days, with gradual shortening of the foam wedge under the sutured wound (promoting bedding down and adhesion of the skin to the underlying tissue) and packing of the lateral cavity with Promogran^TM^ in addition to the VAC foam, as there was still a fair amount of exudate. Skin union of the sutured portion of the wound was noted fairly rapidly after two dressing changes (Figure 15).

**Figure 16:**
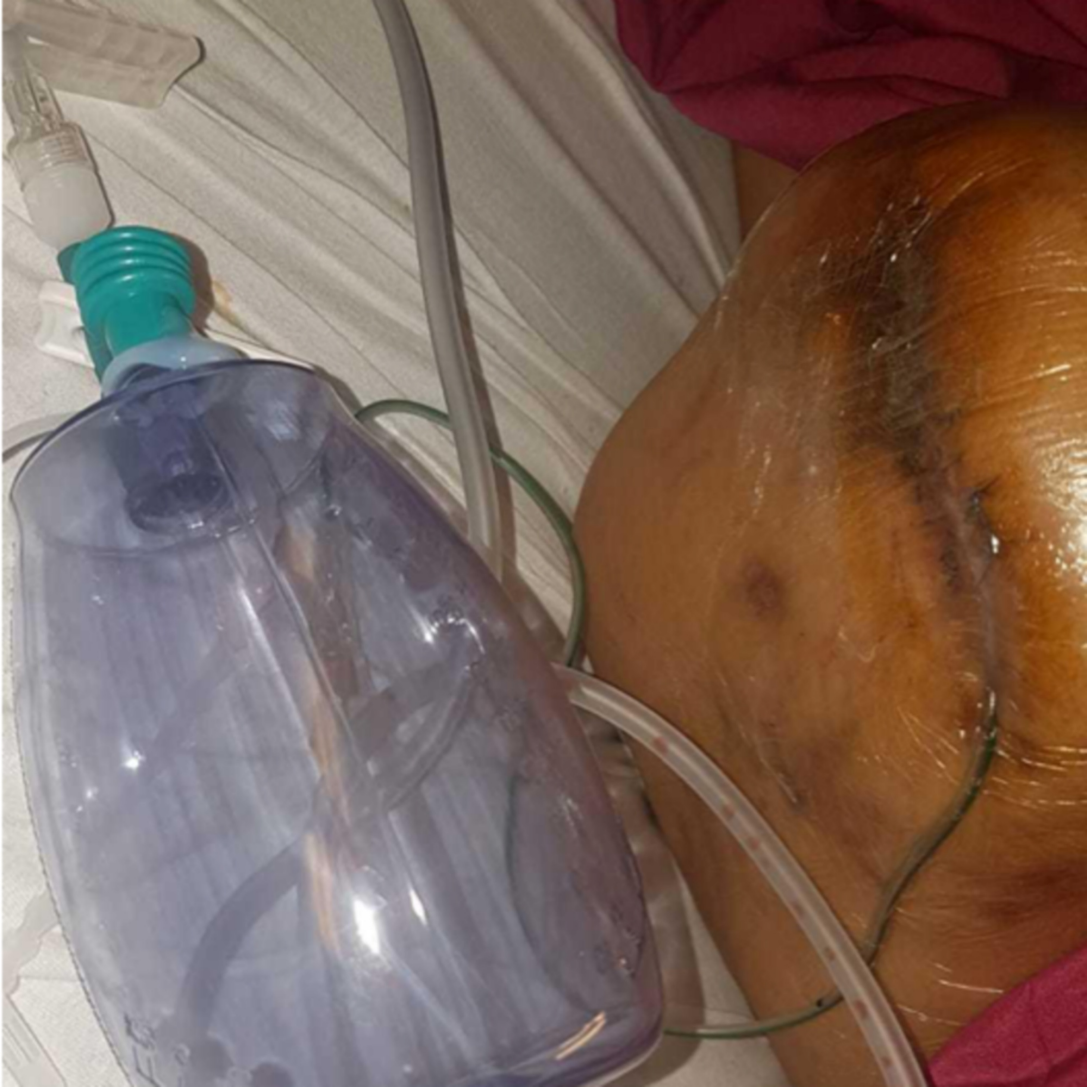
After closure of the wound, with Radivac drain placed between the sutures

Eventually, the remaining 1-inch cavity became less exudative and began contracting, and the lateral wound was secondarily sutured successfully, with placement of a Radivac drain between the sutures (Figure 16). This was removed after fourteen days when drainage stopped. At follow up two months later, the wound had fully healed.

Case 4

A 65-year-old male with a past history of ischemic heart disease, type 2 diabetes mellitus, and end-stage renal failure (on hemodialysis), presented to us with a necrotic right lateral malleolar wound with surrounding cellulitis. An arterial duplex ultrasound scan showed moderate calcification throughout the right lower limb and his toe pressures were 55 mmHg.

Angiography demonstrated an occluded distal SFA with moderate disease in the popliteal artery and occlusion of the PTA trunk, with occlusion of the anterior tibial artery a few centimeters from its origin. The SFA was crossed subintimally and angioplastied with a 6 mm Mustang balloon. The PTA occlusion could not be crossed successfully and during the attempt there was perforation of the PTA trunk with progressive pain and tenseness of the right calf. Hemostasis was secured and an urgent CT peripheral angiogram was performed. This did not demonstrate active contrast extravasation, but showed a large intramuscular hematoma and calf swelling.

He underwent emergency four compartment fasciotomy via medial and lateral approaches and hemostasis. The wounds were pre-closed with loose subcuticular running Prolene stitches and packed with iodine gauze.

**Figure 17:**
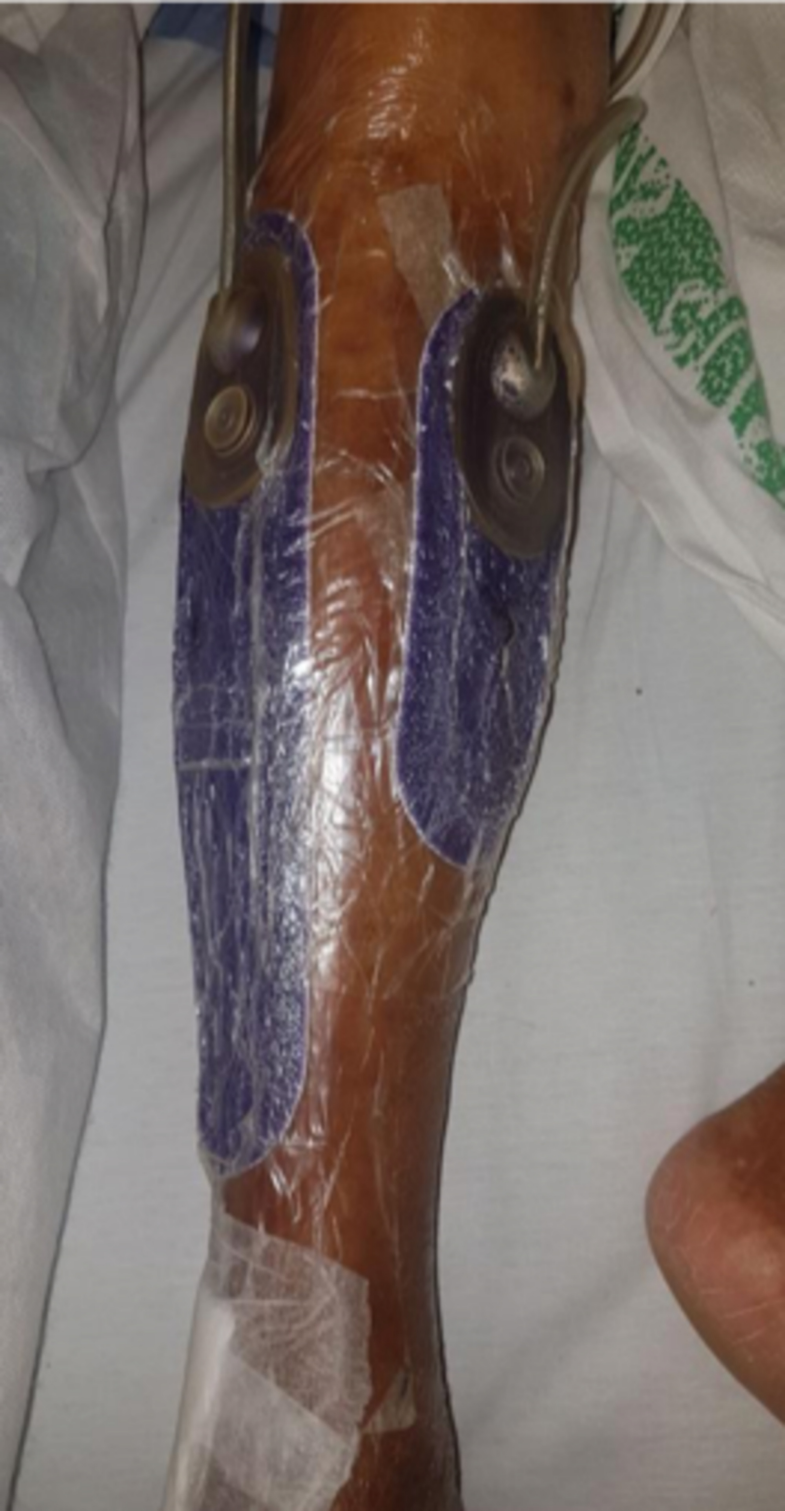
Demonstrating application of Prevena dressings to both fasciotomy incision wounds. Of note, the dressings in the image are not circumferential

After several days, the swelling subsided and the sutures were gradually drawn closed over three days. At this point, a Prevena was placed over each incision as wound prophylaxis, with a Y connector joining both dressings to an ActiVAC machine (Figure 17). Unexpectedly the patient reported marked reduction in pain once negative pressure was applied, and started ambulating almost immediately. He had been complaining of significant wound pain prior to this.

**Figure 18:**
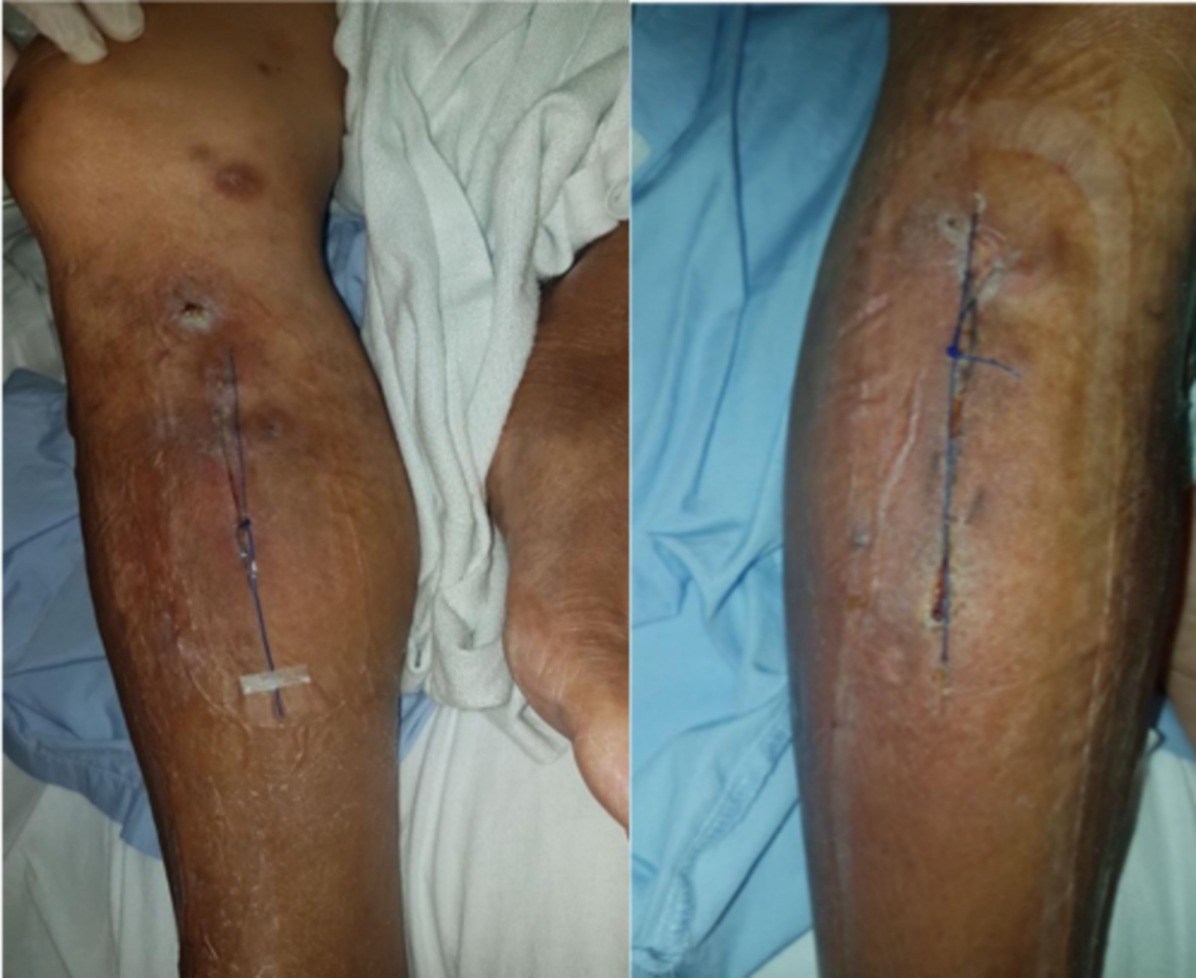
Fully healed wound in clinic a month later

On wound inspection in the clinic a month later, both wounds had healed well (Figure 18).

## Discussion

The main complications of closed surgical incisions are surgical site infection (SSI), wound dehiscence, seroma, hematoma, delayed healing and/or poor scarring. Biomechanical studies analyzing the mechanism of ciNPT have shown it can normalize stress distributions around the closed incision of the skin to homeostatic levels and directions, as well as facilitate enhanced apposition of incision lines, which decreases the risk of wound dehiscence [10]. Moreover, increased lymphatic clearance from ciNPT can reduce the risk of hematoma and/or seroma formation.

The most recent meta-analysis conducted by Hyldig et al. in 2016 demonstrated from 10 randomized controlled trials that ciNPT reduces the rate of the following complications when compared to standard postoperative dressings: wound infection, seroma formation and wound exudate. In general, the most significant outcome studied was that of SSIs, which were decreased by half when ciNPT was used. However, most of the evidence was limited by small sample size and short duration of follow-up [11]. A consensus of the studies to date was published by the World Union of Wound Healing Societies concluding that there is increasing evidence on the benefits of ciNPT on wound complications, as well as a reduction of hospital stay and readmission rate. It also had the potential to lower overall costs by decreasing the need to treat the wound complications [12].

Later, a medical technology innovation briefing issued by the National Institute for Clinical Excellence (NICE) in February 2019 concluded from seven studies that Prevena is more effective at reducing complications than standard care in people with closed surgical incisions [13]. All specialists also agreed that cost savings would likely occur due to reduced need for daily dressing changes. However, a small amount of training would also be needed before implementation of Prevena. In comparison to Pico, which works by a similar mechanism, two specialists mentioned that Prevena was more beneficial as it uses a higher negative pressure of 125 mmHg (as compared to 75 mmHg) and has a thick foam dressing. To date, it is the first and only negative pressure medical device that has a Food and Drug Administration (FDA) indication to reduce the risk of SSIs. Meanwhile, more randomized controlled trials with larger sample sizes are still being carried out to evaluate the benefit of ciNPT in patients with higher risk for wound complications [14].

Use in eccentric wounds

In the first case, Prevena required creative customization as the wound was small and eccentrically shaped on a terminalized big toe which was bridged to the dorsum of the foot. The length of the incision was measured and cut out exactly on the customizable dressing, then trimmed to an adequate width according to the incision with some excess for bridging of the TRAC pad. Hydrocolloid strips were applied to the ends of the incisions and the cut customizable dressing was applied starting from the plantar side of the toe, then around the toe and to the dorsal part of the foot. We focused on ensuring that the webspace of the first and second toe was sealed completely with the sticky hydrocolloid before securing the dressing with the drapes and applying the Prevena TRAC pad on top.

A bridging technique has been described for wounds of the foot in order to avoid complications from pressure injuries mediated by the tubing. On occasion for particularly small wounds, another layer of VAC foam is applied over the drapes before being connected to the TRAC pad. Hydrocolloid dressings have also been found to be useful in obtaining airtight seals in hair-bearing or sweaty regions as it adheres more strongly [15].

Use in hematoma drainage

As mentioned previously, the use of Prevena has been shown to decrease the risk of hematoma and seroma formation [10]. Risk factors for developing such collections include significant tissue disruption or increased dead space, transection of lymphatic channels, and inadequate hemostasis [11]. A randomized controlled trial by Stannard et al. in 2006 showed that negative pressure therapy over a surgical site hematoma had significantly shorter drainage time as compared to pressure dressing [7]. This is of particular relevance since groin incisions are at a higher risk of wound complications, especially SSIs [16]. As a result, vacuum-assisted therapy has been suggested for prophylaxis of a groin wound after vascular surgery. Further meta-analysis in 2020 specific to groin infections showed that negative pressure therapy reduced SSIs, but no other meta-analyses on other outcomes could be performed [17].

In this case, Prevena was used to manage persistent fluid drainage from a liquefying groin hematoma where skin folds and moisture made use of conventional dressings and even stomas unsatisfactory. The high-quality adhesive and negative pressure of Prevena enabled the dressing to stay in place effectively and constantly remove fluid in a closed fashion. It may also have had a role in reducing the chance of developing a secondary infection of the groin hematoma. As compared to normal VAC devices, Prevena has an additional 2.5 cm of foam filler that helps give uniform pressure across the wound. A prospective study also showed previously that ciNPT has a significantly faster wound healing time as compared to open negative pressure therapy [18]. We hypothesize that there may be potential benefit for groin wounds with chronically draining seromas after vascular surgery.

Prevena and VAC combination

VAC dressings are often useful for infection control due to their mechanisms of fluid drainage and mechanical stimulation. However, this is often complicated by fluid collections in hidden spaces between deeper tissues even after VAC is applied. These collections may be out of reach for the VAC therapy to drain effectively, and can be a source of bacterial infection. A previous study has suggested placement of VAC foam between the tissues for better functional reach of VAC therapy [19]. This allows for a minimally invasive surgical exposure for debridement, and may lessen the requirement for post-operative wound coverage.

We combine the concept of a deep cavity VAC dressing with that of Prevena in a previously dehisced AKA wound in this case. The deep cavity VAC helps to drain exudate and possible collections within the potential space beneath the wound, while the exterior layer of Prevena works as ciNPT to decrease risk of infection, dehiscence and seroma formation. The wound eventually healed up fully, which raises the prospect of using ciNPT in tandem with intra-cavity VAC dressings for partially closed wounds with deeper cavities.

Analgesic effects

This case suggests that Prevena provided an analgesic effect when used in lower limb fasciotomy. The use of Prevena was associated with a decrease in post-operative pain, possibly through post-operative edema control to reduce tension on the incision [20]. A randomized controlled trial where Prevena was used on post-cesarean section incisions of patients with body mass index (BMI) >35 showed that besides reducing surgical site occurrences, significantly fewer patients experienced incisional pain at rest and with pressure, leading to a 30% decrease in total opioid use [8]. These findings concur with our case. We postulate external splinting of the sutured wound due to the drape combined with negative pressure therapy reduces shearing forces and tension at the wound edges even during weight bearing. However, this warrants a larger study to ascertain if the effect can be replicated between patients for the benefits to further justify the costs.

## Conclusions

These four cases describe relatively novel uses of ciNPT with good results. We suggest that the paradigm of ciNPT may be expanded to include small wounds and non-resolving hematomas, and can be used in tandem with intra-cavity VAC dressings for drainage of partially closed wounds as there appear to be potential benefits. Initial cost-effectiveness studies already suggest that TNPWT may cost less than conventional dressings for complicated wounds, though further research is needed to evaluate the overall cost-effectiveness of these applications of negative pressure wounds and incisional management.

## References

[1] Kent KC, Bartek S, Kuntz KM, Anninos E, Skillman JJ (1996). Prospective study of wound complications in continuous infrainguinal incisions after lower limb arterial reconstruction: incidence, risk factors and cost. Surgery.

[2] Wallaert JB, Nolan BW, Adams J, Stanley AC, Eldrup-Jorgensen J, Cronenwett JL, Goodney PP (2012). The impact of diabetes on postoperative outcomes following lower-extremity bypass surgery. J Vasc Surg.

[3] Wiewiorski M, Barg A, Hoerterer H, Voellmy T, Henninger HB, Valderrabano V (2015). Risk factors for wound complications in patients after elective orthopedic foot and ankle surgery. Foot Ankle Int.

[4] Davis FM, Sutzko DC, Grey SF (2017). Predictors of surgical site infection after open lower extremity revascularization. J Vasc Surg.

[5] Webb LX, Pape HC (2008). Current thought regarding the mechanism of action of negative pressure wound therapy with reticulated open cell foam. J Orthop Trauma.

[6] Ferrando PM, Ala A, Bussone R, Bergamasco L, Actis Perinetti F, Malan F (2018). Closed incision negative pressure therapy in oncological breast surgery: comparison with standard care dressings. Plast Reconstr Surg Glob Open.

[7] Stannard JP, Robinson JT, Anderson ER, McGwin G Jr, Volgas DA, Alonso JE (2006). Negative pressure wound therapy to treat hematomas and surgical incisions following high-energy trauma. J Trauma.

[8] Gunatilake RP, Swamy GK, Brancazio LR (2017). Closed-incision negative-pressure therapy in obese patients undergoing cesarean delivery: a randomized controlled trial. AJP Rep.

[9] Kwon J, Staley C, McCullough M (2018). A randomized clinical trial evaluating negative pressure therapy to decrease vascular groin incision complications. J Vasc Surg.

[10] Wilkes RP, Kilpad DV, Zhao Y, Kazala R, McNulty A (2012). Closed incision management with negative pressure wound therapy (CIM): biomechanics. Surg Innov.

[11] Hyldig N, Birke-Sorensen H, Kruse M (2016). Meta-analysis of negative-pressure wound therapy for closed surgical incisions. Br J Surg.

[12] World Union of Wound Healing Societies Consensus Document. (2016). Closed surgical incision management: understanding the role of NPWT. Wounds International.

[13] National Institute for Health and Care Excellence (2020). National Institute for Health and Care Excellence: Prevena incision management system for closed surgical incisions (MIB173). Medtech innovation briefing [MIB173].

[14] Rezk F, Åstrand H, Acosta S (2019). Incisional negative pressure wound therapy for the prevention of surgical site infection after open lower limb revascularization - rationale and design of a multi-center randomized controlled trial. Contemp Clin Trials Commun.

[15] Singh S, Mackey S, Soldin M (2008). VAC it - some techniques on the application of VAC dressings. Ann R Coll Surg Engl.

[16] Dosluoglu HH, Loghmanee C, Lall P, Cherr GS, Harris LM, Dryjski ML (2010). Management of early (<30 day) vascular groin infections using vacuum-assisted closure alone without muscle flap coverage in a consecutive patient series. J Vasc Surg.

[17] Sexton F, Healy D, Keelan S, Alazzawi M, Naughton P (2020). A systematic review and meta-analysis comparing the effectiveness of negative-pressure wound therapy to standard therapy in the prevention of complications after vascular surgery. Int J Surg.

[18] Frazee R, Manning A, Abernathy S (2018). Open vs closed negative pressure wound therapy for contaminated and dirty surgical wounds: a prospective randomized comparison. J Am Coll Surg.

[19] Shin SH, Park IK, Kang JW, Lee YS, Chung YG (2018). Vacuum-Assisted Closure (VAC) using multiple pieces for hidden space drainage through less exposure in musculoskeletal infections. J Hand Surg Asian Pac Vol.

[20] Wrotslavsky P (2018). Pain reduction with negative pressure on surgical site incisions. Surg Technol Int.

